# Editorial: Spirituality in the workplace

**DOI:** 10.3389/fpsyg.2023.1162716

**Published:** 2023-06-08

**Authors:** Francesco Chirico, Daniela Acquadro Maran, Manoj Sharma

**Affiliations:** ^1^Post-graduate School of Occupational Health, Università Cattolica del Sacro Cuore, Rome, Italy; ^2^Department of Psychology, University of Torino, Torino, Italy; ^3^Department of Social and Behavioral Health, School of Public Health, University of Nevada, Las Vegas (UNLV), Las Vegas, NV, United States; ^4^Department of Internal Medicine, Kirk Kerkorian School of Medicine at UNLV, University of Nevada, Las Vegas (UNLV), Las Vegas, NV, United States

**Keywords:** worksite, workplace spirituality, spiritual wellbeing, psychology, occupational health psychology, health

Spirituality refers to a feeling of connection between individuals and the universe, meaning in life, purpose, integration, growth, truth, and mindfulness (Harrington et al., 2004). Besides, workplace spirituality has incorporated some spiritual concepts, including inner life connection, sense of community, connectedness, compassion, transcendence, and mindfulness in the workplace, for better mental health and wellbeing of the workers, improved commitment and motivation, and enhanced organizational outcomes of the companies (Beheshtifar and Zare, 2013). Spirituality-based programs in the workplace indeed have been shown to have numerous benefits for employees and organizations, including increased creativity and profits, higher levels of productivity and performance, reduced absenteeism, and improved job satisfaction and intrinsic motivation (Pouragha et al., 2022).

This Research Topic aimed to investigate the connection between occupational health psychology and spirituality to understand better how to develop spirituality-based programs in the workplace. The literature recognizes that spiritual wellbeing is negatively associated with poor mental health outcomes, such as anxiety, depression, and burnout, in helping professionals like teachers and nurses, among others (Chirico and Magnavita, 2019). On the other hand, spiritual wellbeing might predict positive psychological outcomes at work (Chirico, 2017; Sharma, 2018; Chirico et al., 2020). Therefore, spirituality-based programs can reduce burnout, anxiety, depression, and job-related stress and, at the same time, enhance self-career management, organizational self-esteem, and ethical behavior.

In this Research Topic, Yadav et al. showed that employee wellbeing in police personnel is a function of workplace spirituality, empathic concern, and organizational politics. The empirical study by Liang et al. among scientific and technological workers in China has highlighted that meaning of work has a positive impact on innovative behaviors. Garg et al. have investigated the “necessity and sufficiency of gratitude for supporting workplace happiness among Indian university teachers.” The authors found that gratitude is a sufficient and necessary condition for workplace happiness. Furthermore, they discovered a significant mediating effect of psychological and social capital in the relationship between gratitude and workplace happiness. Sousan et al. showed the adverse impact of practicing surface acting (SA) on the mental health of Iranian nurses confronting COVID-19. Moreover, they showed the buffering effect of the sense of community on the relationship between SA and job stress. Finally, Bella et al. analyzed “*An initial approach to increase job satisfaction through workplace spirituality*.”

The findings of these studies suggest that health planners and authorities should consider some factors that contribute to the promotion of workplace spirituality to increase the motivation of the workers for improved performance and organizational wellbeing of the companies. For example, the COVID-19 pandemic revealed a connection between mental health, spirituality, and fear (Chirico, 2021). Spiritual skills can be special tools for healthcare workers during global emergencies like SARS-CoV-2 and future challenges posed by climate change and global disasters (Chirico and Nucera, 2020). Finally, spiritual skills may be helpful to cope with adversity and workplace issues (Magnavita and Chirico, 2020), especially for the helping professions and in end-of-life and palliative care settings. Such development of skills related to spirituality must be based on evidence-based (behavioral theory-based) research (Sharma, 2022). Third-generation theories such as the health belief model, social cognitive theory, theory of planned behavior, and others, as well as fourth-generation theories such as the multi-theory model (MTM) of health behavior change, integrative model of behavioral prediction and others, can be useful in this direction (Sharma, 2022).

Workplace spirituality can serve as a framework for promoting employee wellbeing by manifesting organizational values and culture. Furthermore, by incorporating spirituality into workplace health promotion programs, employees can be supported by their organizations to cope with burnout, work-related stress and violence, and other psychosocial occupational risk factors. For this reason, employers should consider implementing spirituality-based programs in the workplace to reap the numerous benefits they can offer. In addition, scholars should better understand the relationship between burnout, stress-related disorders, and the spiritual wellbeing of the workers, as well as the effectiveness of meditation, yoga, and other spirituality-based activities at the workplace.

## Author contributions

FC: writing—original draft preparation. MS and DA: writing—review and editing. All authors have read and agreed to the published version of the manuscript.
